# An overview of nursing and midwifery leadership, governance structures, and instruments in Africa

**DOI:** 10.1186/s12912-023-01336-3

**Published:** 2023-05-18

**Authors:** Faith Nawagi, Rosie Kneafsey, Mohammed Modber, Madeline Mukeshimana, Cecilia Ndungu, Lisa Bayliss-Pratt

**Affiliations:** 1 College of Health Sciences, Makerere University, Kampala, Uganda; 2 Centre for Healthcare Research, Coventry University, Coventry, UK; 3 Médecins Sans Frontières, Khartoum, Sudan; 4 College of Medicine and Health Sciences, University of Rwanda, Kigali, Rwanda; 5 The National Nurses Association of Kenya, Nairobi, Kenya; 6 Nursing Now Challenge, Coventry, UK

**Keywords:** Nursing, Midwifery, Governance, Structures, Instruments, Africa

## Abstract

**Background:**

Investment in nursing and midwifery leadership and governance are key suggested approaches by the World Health Organization (WHO) Africa Regional Office to address the shortages in the nursing health workforce. However, there are few if any studies that have investigated the existence and operationalization of the nursing and midwifery leadership and governance structures in Africa. This paper fills this gap by, providing an overview of nursing and midwifery leadership, governance structures, and instruments in Africa.

**Methods:**

We conducted a descriptive cross-sectional study of the nursing and midwifery leadership, structures, and instruments in 16 African countries using quantitative methodology. Data was analyzed using SPSS IBM 21 statistical software. Data was summarized in frequencies and percentages and presented as tables and charts.

**Results:**

Only (9,56.25%) of the 16 countries included had retrievable evidence of all expected governance structures while (7, 43.75%) lacked one or more of the structures. A quarter (4, 25%) of the countries did not have a department of nursing and midwifery or chief nursing and midwifery officer at their Ministry of Health (MOH). The dominant gender representation across all the governance structures was female. Only Lesotho (1, 6.25%) had all expected nursing and midwifery governance instruments while the remaining (15, 93.75%) had either one or four of these instruments missing.

**Conclusions:**

The lack of complete nursing and midwifery governance structures and instruments in various African countries is a matter of concern. Without these structures and instruments, the strategic direction and input of the nursing and midwifery profession cannot be maximized for the public good in relation to health outcomes. Addressing the existing gaps requires a multipronged approach with the need to strengthen regional collaboration, and advocacy, creating awareness, and advancing nursing and midwifery leadership training to enable nursing and midwifery governance capacity development in Africa.

**Supplementary Information:**

The online version contains supplementary material available at 10.1186/s12912-023-01336-3.

## Introduction 

Advancement of nursing and midwifery leadership remains a global impetus suggested by the recent 2020 State of the World’s Nursing report by the World Health Organization (WHO) [1]. The Nursing Now Challenge, previously known as the Nightingale Challenge has played a key role in raising the status of early-career nurses and midwives and generating leadership opportunities around the world with significant outcomes [2].

In 2022, a study was facilitated by the Nursing Now Challenge to map and document the existence or absence of nursing and midwifery leadership, governance structures, and instruments in Africa. Funded by the Burdett Trust for Nursing this work has provided an important starting point in identifying ways to harness and maximize nursing and midwifery leadership within Africa as a means of improving population outcomes. This paper reports the findings of the study.

There is relatively little evidence on the dynamics of nursing and midwifery leadership in Africa. However, there is a clear indication of nurses and midwives being less involved in health policy development in the sub-Saharan African countries included in this study [3]. This is largely attributed to the tendency for health sector leadership and policy decision-making to be led mainly by doctors globally including in Africa [4]. Furthermore, nurses and midwives in Africa often lack effective advocacy skills, mainly attributed to a lack of leadership training included in the nursing and midwifery curriculum at bachelor’s, diploma, and certificate levels [5]. The profession, whilst being female-dominated is also affected by gender inferiority where many times women are under-represented in health leadership positions [6]. This gender inferiority has created a challenging context for the nursing and midwifery professional bodies that govern the professions to advance and advocate for nurse and midwife involvement in key health sector policies and decisions. Yet it is nurses and midwives that serve on the frontline and typically have first-hand evidence to guide policy decisions.

Nursing and midwifery governance structures are the bodies or parastatals (state-owned organizations with political authority) put in place to run the functions of the profession [7]. The nursing and midwifery governance instruments are the tools used by each governance structure to guide its operations [8]. Effective leadership in nursing and midwifery globally and in Africa takes place through the existence of various governance structures [9] that include; a department of nursing and midwifery at the Ministry of Health (MOH), a nursing and midwifery regulatory body, education and examination body, nursing and midwifery professional associations, and nursing and midwifery labor unions [9]. The governance structures are guided by various governance instruments. These include [2] nursing and midwifery;

**Scope of practice** that highlights the role of each nursing and midwifery cadre based on their training level and license in a particular country [2].

**Schemes of service** that guide the deployment of nurses and midwives in the various health care centers [2].

**Nursing and midwifery policy** which is a set of overarching principles and goals that guides the various sectors of nursing and midwifery which include service and health care, education, regulation, research, ethical conduct, and quality assurance, among others [2].

**Strategic plan** which guides the implementation of the policy within a given time frame. It sets out a vision for the future and identifies goals and objectives to be achieved through a well-structured implementation approach [2].

**Codes of conduct and ethics** guide the professionalism as nurses and midwives perform their roles [10].

While the above nursing and midwifery governance structures and instruments are key in advancing nursing and midwifery leadership, little is known about their existence in Africa [11]. Furthermore, little is known about the state of their operationalization, and emphasis on promoting nursing and midwifery leadership skill acquisition.

This research study addressed this gap by mapping the state of nursing and midwifery leadership, governance structures, and instruments in Africa.

## Methodology

### Study method

This was a descriptive cross-sectional study using quantitative methodology. This involved the online collection of data from each country's representative, using an online survey at one point in time. Data was then quantified using frequencies and proportions to establish the state of nursing and midwifery leadership, governance structures, and instruments in Africa. All methods were performed in accordance with the relevant guidelines and regulations.

### Study setting

This project was facilitated via the Nursing Now Challenge which is a global campaign that works with health employers as well as universities and colleges around the world to create leadership development opportunities for early-career nurses and midwives [12]. This global network engages nurses and midwives in development activities through its Challengers' Committee which is made up of six Regional Hubs (Africa, Americas, Eastern-Mediterranean, Europe, South-East Asia, Western Pacific). Members of the Challengers’ Committee work with the Nursing Now Challenge team to identify innovative ways to support nurses and midwives in their roles as leaders, practitioners, and advocates in health. Their work helps to demonstrate that nursing and midwifery are exciting and rewarding careers that contribute to improved recruitment and retention on a global scale.

Each of the Challengers’ Committee Hubs is chaired by two members of the Committee and has members. The Africa hub has various members from different countries. These countries are (17); Botswana, Burundi, Cameroon, Ethiopia, Ghana, Kenya, Lesotho, Malawi, Namibia, Nigeria, Sierra Leone, South Africa, Sudan, Tanzania, Uganda, Zambia, and Zimbabwe. Most of the activities of the hub are done online due to COVID-19 but also due to cost-effectiveness in delivery and running activities with the online approach. The Africa regional hub is a virtual hub and currently has 25 members as per the different countries listed above. These nurses and midwives are actively practicing in various leadership and frontline roles in their respective countries.

### Study population

The study population for this study was the nurses and midwives fulfilling the role of country-specific representatives and members of the Nursing Now Challenge, Challengers’ Committee, and African regional hub. These nurses and midwives were those living and practicing in their country in the hospital, research, academic setting, and involved in various nursing and midwifery advocacy activities. Furthermore, the participants were those who had valid licensure in their country at either diploma, bachelor's, or master's level of training. Each of these members was given an online survey to effectively search for related information on the nursing leadership, governance structures, and instruments i.e. policies, strategic plans, the scope of practice, and schemes of service specific to their country.

### Sampling method and recruitment of participants

Purposive sampling was used in this study to enable the detailed collection of data per country from each country's Challengers’ Committee Hub representative since the scope to sample from our study setting was limited. It required one person to be the focal person given the limited time frame and funding. Only members of the Africa regional hub who had time to commit to this piece of work were recruited. Members who could not provide consent or meet the project timeline were not included.

### Study tools

All tools were administered in English and no translation was required since the study targeted the literate community and English is used as the official training language for the nursing profession in all the African regional hub member countries participating except Burundi. However, the member from Burundi is fluent in English.

### Data collection tool

The data collection tool was developed as a checklist with open and closed-ended questions to enable capturing of the specific details required regarding each variable being studied. This was reviewed by the study team for effectiveness. This tool was developed by the research team simply because no tool has been developed for this purpose in Africa at large. The first section involved questions on the nursing and midwifery governance structures and leadership existence at each structure i.e., the Department of Nursing and Midwifery at the MOH, Regulatory Body, Education and Examination Body, Nursing and Midwifery Union, and Nursing and Midwifery country wide Association coupled with understanding the various levels of nursing and midwifery training in each country. The second section involved questions on the various nursing and midwifery governance instruments i.e., the nursing/midwifery policy, strategic plan, nursing/ midwifery scope of practice, nursing/ midwifery schemes of service, and code of conduct and ethics. The questions sought to understand the years of development and if they are currently operational. The third section involved understanding the differences in professional governance of nursing and midwifery, practice differences at the front line, addressing the gaps, causes of the gaps in nursing and midwifery governance, coupled with suggestions on how to address the gaps. The detailed tool is attached as a supplementary file.

Team members were asked to find evidence of the existence of the named structures, either hard or online copy based on the assumption that such structures and instruments would be publicly available and transparent to wider society, the professions of nursing and midwifery, and the general population.

### Data collection

Data collection was done online from 21^st^ March to April 30^th^ 2022. The members who collected the data underwent orientation training to enable a uniform understanding of all the constructs in the tool being used. Each country representative submitted details about their country via Microsoft Forms and inserted a reference for each of the details. Each country representative was given instructions on how to search for and obtain the data being sought. This required each Hub member to contact their various nursing and midwifery governance structures, and their websites, and undertake a Google search. Questions on the operationalization of the instruments required the data collection person per country to contact the leaders or the key staff at each of the structures. Furthermore, a literature search on all the nursing and midwifery governance structures and instruments being studied was completed by a Coventry University librarian via Cinahl a research database, and a range of search engines, suited to the discovery of gray literature. Details on each document were captured and the PDFs available were downloaded and uploaded in the Microsoft forms. Each member needed to state the source of the data and provide a web link if obtained online. For those countries where data was obtained offline and accessed as hard copies, the researcher stated the source of the hard copy and upload it in Microsoft Forms. The Data collected was then downloaded as a data set, cleaned, and fed to SPSS 21 IBM statistics for data analysis.

### Data analysis

Data was cleaned before being subjected to analysis. Data analysis was performed using SPSS 21 IBM statistical software [13]. Information was summarized using frequencies and percentages and presented in frequency tables, and figures.

## Results

This section describes the findings from this study in line with existing nursing and midwifery leadership, governance structures, and instruments in Africa. Seventeen members of the Challengers’ Committee, Africa Hub, each from a different country, agreed to actively participate in the study. Out of the 17, 16 members were able to return their country profile securing a 94% response rate.

### Existence of nursing and midwifery governance structures in various African Countries

As shown in Table 1, only nine (56.25%) of the countries had all the nursing and midwifery structures while seven (43.75%) lacked one or more of the structures. Burundi and Sudan had the least evidence of visible nursing and midwifery leadership structures, followed by Ethiopia and Rwanda. Tanzania, South Africa, and Sierra Leone lacked only one key structure i.e. the nursing and midwifery labor union.

**Table 1:** Country distribution of nursing and midwifery governance structures in various African countries. *N* = 17

Country	Department of Nursing/Midwifery	Chief Nurse /Midwife at the MOH	Nursing / Midwifery Regulatory body	Nursing /Midwifery Examination body	Nursing and Midwifery Labor Union	Nursing and Midwifery Federation/ Association
Uganda	X	X	X	X	X	X
Kenya	X	X	X	X	X	X
Malawi	X	X	X	X	X	X
Botswana	X	X	X	X	X	X
Zambia	X	X	X	X	X	X
Namibia	X	X	X	X	X	X
Lesotho	X	X	X	X	X	X
Ghana	X	X	X	X	X	X
Nigeria	X	X	X	X	X	X
Tanzania	X	X	X	X		X
South Africa	X	X	X	X		X
Sierra leone	X	X	X	X		X
Rwanda			X		X	
Ethiopia				X		X
Burundi						
Sudan						

X means that the country has that structure present while the empty box means no structure is present

As shown in Table 2, a quarter (4,25%) of the countries did not have a department of nursing/ midwifery or chief nursing/ midwifery officer at their MOH. Nevertheless, the most dominant gender of representation across all the governance structures was female as shown in Table 1 except for the national nursing and midwifery association which had more male leadership (9, 69%). The majority of the countries, (13,81%) reported having a nursing and midwifery regulatory body. All countries provided nurse and midwifery training from certificate level (the lowest) through to Ph.D. level (highest) with Diploma level (15,93.75%) and Bachelors level (16,100%) being the most dominant. However, despite the existence of the various levels of training, (11,68.75%) of the countries do not have a difference in nursing/midwifery roles in day-to-day practice at the frontline in the various hospitals. The majority of the countries have country-wide nursing and midwifery associations (13, 81%), though almost half (6,46%) of these associations were not considered active by the respondents in influencing nursing and midwifery policy or nursing or midwifery agendas in their countries. More than a quarter (6, 38%) of the countries did not have a labor union while (12,75%) lacked official nursing and midwifery leadership training for their leaders and nurses and midwives in practice. It is important to note that the majority (11, 68.75%) of the countries utilized a governance structure that catered to both nursing and midwifery professions.

**Table 2:** Frequency and percentage distribution of nursing and midwifery governance structures in various African countries. *N* = 16

Variable	Frequency (n)	Percentage (%)
Dept of Nursing/Midwifery		
Yes	12	75
No	4	25
Chief Nurse/Midwife		
Yes	12	75
No	4	25
Gender of chief Nurse /Midwife		
Female	11	
Male	1	
Regulatory Council/ body		
Yes	13	81
No	3	19
Gender of Registrar of the Council		
Female	9	69
Male	4	31
Various Training levels		
Certificate	11	68.75
Diploma	15	93.75
Bachelors	16	100
Masters	14	87.5
PHD	10	62.5
Nursing/Midwifery Education and Examination Body		
Yes	13	81
No	3	19
Gender of the leader of the Examination Board		
Female	6	54
Male	7	46
Nursing and Midwifery Country-wide Association		
Yes	13	81
No	3	19
Gender of the leader of the Association		
Female	4	31
Male	9	69
Active in Influencing policy		
Yes	7	54
No	6	46
Nursing and Midwifery Labor Union		
Yes	10	63
No	6	38
Gender of Labor Union Lead		
Female	12	75
Male	4	25
Official Leadership training for all Nurse/Midwife Leaders		
Yes	4	25
No	12	75
Necessary to have leadership training		
Yes	16	100
Nursing and Midwifery are governed as the same profession with the same bodies		
Yes	11	68.75
No	5	31.25
At the frontline in daily Hospital Practice, there is no difference in roles based on education. It’s the same roles provided by all nurses		
Yes	11	68.75
No	5	31.25

### Existence of nursing and midwifery governance instruments in various African countries

The nursing and midwifery governance instruments that we searched for included the nursing and midwifery, policy, strategic plan, scope of practice, schemes of service, and codes of conduct. As shown in Table 3, only one country (Lesotho) had all the nursing and midwifery governance instruments in place while all the other countries (15,93.75%) had either one or four of these instruments missing.

**Table 3:** Country distribution of the existence of the nursing and midwifery governance instruments in various African countries. *N* = 16

Country	Nursing and Midwifery Policy	Nursing / Midwifery Strategic Plan	Nursing / Midwifery Scope of Practice	Nursing / Midwifery Schemes of Service	Nursing and Midwifery Codes of Ethics
Uganda	X	X		X	X
Kenya	X		X	X	X
Rwanda			X		X
Sudan			X		X
Ethiopia					X
Tanzania			X		X
Burundi					
Malawi	X	X	X		X
Zambia	X	X	X		X
Botswana	X	X	X		X
Namibia	X	X	X		X
Lesotho	X	X	X	X	X
South Africa	X	X	X		X
Ghana	X	X	X		X
Nigeria	X			X	X
Sierra leone	X	X		X	X

X means that the country has that governance instrument present while the empty box means no governance instrument is present

As shown in Table 4.

**Table 4:** Frequency and percentage distribution of the nursing and midwifery governance instruments in various African countries. *N* = 16

Nursing/ Midwifery Governance Instruments	Mean Years since the development	95% CI	Available to the Public n(%)	Effectively operational on ground n(%)
**Nursing/Midwifery Policy**	17 (± 15.5)	-.325- 22.8		
Yes 11 (68.75)			Yes 9 (82)	Yes 9 (82)
No 5(31.25)			No 2 ( 18)	No 2 (18)
**Nursing/Midwifery Strategic Plan**	3.5 (± 3.7)	-.92–12.4		
Yes 9 (44)			Yes 6(67)	Yes 3 (33)
No 7 (56)			No 3(33)	No 6 (67)
**Nursing/Midwifery Schemes of Service**	4.5 (± 2.9)	-.093–9.0		
Yes 5 (31%)			Yes 4 (80)	Yes 2 (40)
No 11 (69%)			No 1 (20)	No 3( 60)
**Nursing/Midwifery Scope of Practice**	13.4 (± 14.1)	1.5–11.4		
Yes 11(68.75)			Yes 10 (91)	Yes 9 (82)
No 5 (31.25)			No 1(8)	No 2 (18)
**Nursing/Midwifery Codes of Conduct and Ethics**	7.1(± 4.0)	-1.16 -16.1		
Yes 15 (93.75)			Yes 14 (88)	13 (86.6)
No 1 (6.25)			No 1 (12)	2 (13.4)

More than half (11,68.75%) of the countries have a nursing and midwifery policy with the mean years since the development of the current operation policy being 17 years across all countries. However, more than a quarter (5,31.25%) of the countries lack a nursing and midwifery policy for the nursing and midwifery profession. In those countries where the policy is existent (11, 68.75%), the majority (9,82%) have the policy available to the public and operational. However, only (2,18%) have their policy currently operational.

With regards to the nursing & midwifery strategic plan as shown in Table 4, (9, 44%) of the countries have strategic plans with the average years since development being 3.5 years, while more than half (7, 56%) do not have a strategic plan. For those with strategic plans, more than a quarter (3, 33%) are not available to the public and more than half (6, 67%) are not currently operational.

As shown in Table 4, the majority of the countries (11, 69%) lack a scheme of service. However, for those countries with a scheme of service (5, 31%), the average years since the development of the current operational scheme of service is 4.5 years, (4, 80%) are available to the public while more than half (3, 60%) are not effectively operational on the ground.

More than half (11, 68.75%) of the countries have a nursing and midwifery scope of practice with the average years since development for the currently operational one being 13.4 years as shown in Table 4. The majority (10, 91%) of the countries with nursing and midwifery scope of practice are available to the public and only (2,18%) are not effectively operational on the ground.

As shown in Table 4, only one country, Burundi has no codes of ethics and conduct while the rest of the countries (15,93.75%) have codes of conduct and ethics. The average years since the development of the currently operational version is 7.1 years. The majority of the codes of ethics and conduct are available to the public (14, 88%) and (13, 86.6%) are operational.

### Reasons for the absence of the various nursing and midwifery governance structures and instruments

The reasons given by the representatives of the Challengers’ Committee Africa Hub for the lack of nursing and midwifery governance structures and instruments include:

- Lack of a MOH department for nursing/ midwifery to develop the required documents.
- Existence of composite documents that address the entire Human Resources for Health and not having a focus on nursing/midwifery.
- Lack of advocacy skills among some leaders leading the governance structures
- Insufficient financing of the nursing/ midwifery structures from the government
- Lack of awareness among nurses and midwives about the importance of the nursing and midwifery governance structures and instruments.

### Suggested strategies to advance nursing and midwifery governance structures and instruments in various African countries

**Fig. 1:**
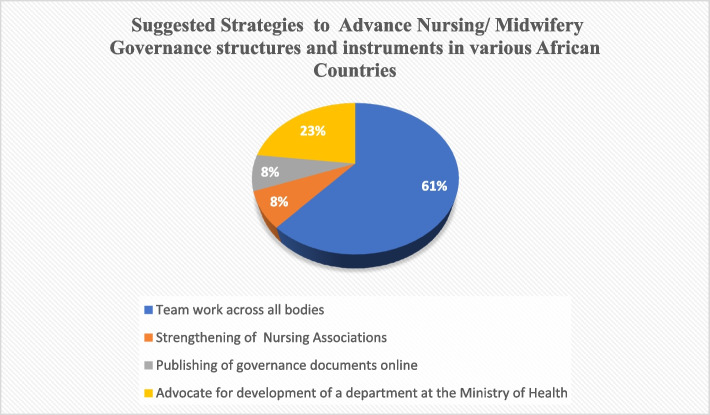
Suggested strategies to advance nursing/midwifery governance structures and instruments in various African countries

We asked the representatives from the Africa Regional Hub what strategies they felt would advance the governance structures of nursing and midwifery. Their suggestions are shown in Fig. 1.

## Discussion

Nursing and midwifery governance across Africa is largely the role of the government. This is evidenced by the findings of this study and the WHO State of the World Nursing 2020 report where most African countries have nursing and midwifery governance structures as part of government institutions [14]. This study shows that nursing and midwifery structures are led by women. This can be attributed to the fact nursing and midwifery are female-led professions in Africa [15]. Nursing and midwifery leadership roles come with the expectation that one should be equipped with adequate skills to run designated tasks, including the development and implementation of governance instruments [16]. Nurses and midwives contribute the largest percentage of health workers on the frontline in Africa and globally [17]. Their ability to have effective leadership and management skills is critical in advancing universal health coverage and in situations of public health security [18].

In this study, three-quarters of the countries reported a lack of nursing and midwifery leadership training for their leaders and nurses. These findings are similar to the findings in the nursing and midwifery leadership predictors study in Uganda [3] and across the region [16] where lack of leadership training among nurses and midwives has posed challenges to health systems and workforce development in Africa [19].

For the effective deployment of the nursing and midwifery profession in any country, there should be a department of nursing/ midwifery at the MOH to lead and develop policies to govern the profession and the nursing and midwifery care given to the population [3]. The nursing /midwifery department should be able to work in unison with other bodies like the professional regulatory body to ensure effective workforce regulation and licensure and work with the body in charge of education to ensure quality education and standardized proficiencies and competencies are clearly established and assessed [20].

In this study, we found that almost a quarter of the countries surveyed lacked a department of nursing/ midwifery a chief nurse/ midwife at the MOH, a regulatory body, and a body in charge of nursing and midwifery education and examination. This gap leads to inadequacy in the advancement of the profession characterized by inadequate planning and structuring of the nursing and midwifery health workforce required to meet the health needs of the population in Africa [21]. This also leads to less representation and involvement of nurses and midwives in key decision-making platforms leading to the nursing and midwifery concerns being less prioritized [16]. In the long run, this leads to nursing and midwifery workforce shortages which directly impede the ability to achieve universal health coverage [20]. A stronger and better-led nursing and midwifery profession largely starts with the existence of the Department of Nursing and Midwifery at the MOH. This is because it is charged with the role of developing the nursing and midwifery policy and strategic plan that guides the nursing and midwifery services, regulation, and education in each country [9]. This eventually determines the quality of health care and health outcomes.

Nursing and midwifery regulation is a key construct in any country to ensure that the population gets quality nursing and midwifery care from a well-trained and registered nurse/ midwife competent to provide the required nursing/midwifery services. Furthermore, it performs regulatory visits to the health facilities, and training facilities to ensure the minimum standards are adhered to [20]. To strengthen regulation and harmonize standards across Africa, development of joint bodies like the African Health Profession Regulatory Collaborative for Nurses and Midwives was developed in partnership with 14 countries to strengthen nursing regulation needs across various African countries in partnership with Emory University [22]. This began in 2011 as a four-year initiative funded by the United States of America Centers for Disease Control and Prevention and ended in 2015. This led to the advancement of leadership development, shared learning, and support across countries for regulatory reforms [23]. It is such initiatives that should be strengthened and expanded to more African countries including the francophone speaking countries to support countries that are lacking regulatory bodies just like those reported in this study.

The most dominant levels of education and training of the nurses/midwives in this study were diplomas and bachelors. These findings are similar to those in the World Bank report in partnership with the East, Central, and Southern Africa College of Nursing (ECSACON) where diploma training was the most dominant form of training [24]. Although the majority of the countries in this study reported the existence of training in nursing/ midwifery at the master's and Ph.D. levels, the most recent World Bank report showed a need for scaling in these levels of training [24]. Nursing and midwifery education across Africa remains an area that needs scaling and harmonization to address nursing and midwifery health workforce shortages across Africa. Although the East African community has made an effort to harmonize training standards across five countries [25], the gap remains broader given that Africa has over 54 countries with 47 being under the WHO AFRO region [26].

The Nursing and midwifery labor union's main role is to advocate for the welfare and rights of nurses and midwives. Their existence is critical in lobbying for better work conditions and remuneration for the nurses and midwives. This enables a collective voice for the nursing and midwifery profession in addition to the nursing and midwifery associations in the various countries that enable collective joint efforts in all matters concerning the profession, including aspects of professionalism. Although these are key, over half of the countries in this study mentioned that both the labor union nor associations were not active in influencing nursing /midwifery policies and advancing the interests of nurses and midwives. This is mainly because many of these are often under-resourced and lack enough financial support to advance their work [27]. Furthermore, since the association and labor union leaders are also nurses/ midwives serving in various nursing/ Midwifery sectors, balancing out time to advance issues is challenging since these roles are often taken on voluntarily on a part-time basis.

The nursing and midwifery governance instruments that were studied included the nursing and midwifery policy, the strategic plan, the scope of practice, schemes of service, and codes of ethics. These instruments enable the nursing and midwifery structures to perform their roles adequately. However, in this study, most of the instruments currently being used had wide mean confidence intervals with some being as old as more than 10 years. These findings are similar to those in a recent report on the state of the health workforce in the WHO Africa region, which also found gaps in governance instruments and structures [28]. The evidence suggests that the existing policy documents, strategy, and related instruments are outdated and no longer represent contemporary thought, knowledge, and best evidence. A future-facing stance must be formulated via which to structure preparations and readiness for potential, actual, or foreseen socio-political, economic, health, and environmental challenges ahead.

The nursing/ midwifery policy is a critical instrument for any country to effectively govern the profession. It ensures all areas of nursing and midwifery are addressed and streamlined for effective nursing and midwifery care for the population. Despite its importance, more than a quarter of the countries in this study lacked a nursing/midwifery policy. These findings relate to many of the challenges of the nursing/midwifery profession mentioned in the region including the significant gaps in maternal and child health services, largely the remit of midwifery [29]. The policy gives direction to the profession and its implementation is guided by the nursing and midwifery strategic plan. However, in this study more than half of the countries lacked strategic plans and almost three-quarters did not have their strategic plans being operational and implemented. These findings are similar to the broader challenges of health workforce governance in Africa and thus undermine the ability to effectively meet the demand for nurses and midwives in the various countries which eventually leads to difficulty in strategic capacity development and financing [30]. The development of the nursing and midwifery policy and strategic plan is mainly a role of the department of nursing/midwifery at the MOH led by the chief nurse/midwife whose absence in some of the countries in this study, explains the lack of nursing/ midwifery governance instruments (Nursing policy and strategic plan).

The nursing and midwifery scope of practice is an important instrument to effectively ascribe roles for nurses and midwives trained at different levels to provide nursing and midwifery care. This is usually dynamic and influenced by nursing and midwifery health workforce shortages, workforce planning by governments, task shifting, and healthcare technology among others [31]. Although most of the countries mentioned have a scope of practice that is effectively operational, the majority of the countries reported that on the frontline in daily hospital practice, there is no difference in roles based on education—the same roles are provided by all nurses/ midwives irrespective of educational background and qualifications. Furthermore, from a desktop review of the various scopes of practice, several countries have only a limited general description of the roles of a registered nurse or registered midwife without a clear explanation of the categories of registered nurses/midwives by the level of training. This points to a weakness in effectively structuring roles in alignment with the level of training which is key in the differentiation of skills and competencies accrued at different levels. This undermines the aspiration of nurses/midwives to upgrade their training since there is no difference in roles in practice and most likely a lack of additional remuneration with increased competence or specialization. Furthermore, these findings point to the weakness of the nursing and midwifery leaders in understanding what roles should be given to the various cadres of nurses and midwives trained at different levels. Despite the gaps, the most recent scope of practice in Kenya highlights the roles of nurses and midwives according to their training levels [32]. It is hoped that this can be effectively implemented at the grassroots level to enable benchmarking by other African countries.

One of the key strengths across almost all countries was the existence of the codes of conduct and ethics for nurses and midwives in this study. These are critical in providing moral and professional guidance and a means of holding nurses and midwives accountable as they perform their roles to meet the healthcare needs of the population, work with other nurses as they perform their roles, and adhere to nurses' and midwives' professional requirements [33].

Despite all the gaps, Lesotho is one country that reported having all the nursing and midwifery governance structures and instruments. This could be attributed to the fact that Lesotho reported the presence of leadership across all the governance structures. However, Lesotho just like the majority of the countries in this study also reports a lack of differentiation in roles for the nurses and midwives at the frontline with all nurses and midwives, irrespective of the level of training doing the same roles at the frontline in various health care facilities.

One of the key ways to generate a change to advance and develop the missing nursing and midwifery governance structures and instruments is through advancing nursing and midwifery leadership training to enable nurses and midwives to gain effective leadership skills like advocacy, decision-making, and development of policies among others [17]. Furthermore, synergies can be harnessed through regional collaborations that enable regional standardization and quality assurance of nursing and midwifery governance through the development of a nursing and midwifery governance accrediting body that works with governments to enforce quality checks and the existence of structures. The starting point could be strengthening the College of Nursing for east,central and southern Africa and the West African College of Nursing to take up this role in these regions and possibly expand to other regions and countries where they are not currently operating.

## Conclusion

This study indicates that the crucial governance structures and associated instruments required for effective nursing and midwifery leadership, development, and strategic workforce deployment are, absent or outdated within many of the 16 African countries sampled herein. Failure to utilize and harness the full potential of these professions in pursuit of improved population health outcomes is a significant negative consequence of this neglect. Addressing the existing gaps requires a multipronged approach with the need to strengthen regional collaboration, advocacy, creating awareness, and advancing nursing/midwifery leadership to enable nursing and midwifery governance capacity development in Africa.

### Recommendations

There is a need to expand the study to other African countries to gain a wider picture of the situation. This would eventually guide the various stakeholders to design interventions that are evidence-based with innovative approaches that are sustainable in the region.

Future studies focusing on the processes of developing nursing and midwifery governance structures and instruments in each country would be beneficial. It would also be valuable to explore the views of African nursing and midwifery leaders on the next steps that are needed to progress nursing and midwifery governance using a sustainable approach. Lastly, understanding the nursing and midwifery styles in each country would also be beneficial and key in promoting benchmarking in the region. While this study focused on providing an overview of the existence of the various nursing and midwifery governance structures and instruments, a detailed document analysis of the various policy documents and identify key themes and patterns related to nursing and midwifery leadership and governance in Africa remains key and is being done by the study team.

### Quality control

Face and content validity was done to ensure that the questions being used were adequate to measure the constructs being studied. Furthermore, a reliability test using Cronbach alpha was done and a result of 0.72 was obtained and considered acceptable. Piloting of the tools was done internally by the Nursing Now Challenge team in the UK and if anything needed to be adjusted, it was done to enable accurate and adequate data collection.

### Limitations

This study only reports results from 16 countries from predominantly anglophone-speaking countries. This was largely due to the recruitment of only countries active in the Nursing Now Challenge in Africa. This leaves a gap in understanding the nursing and midwifery governance structures and instruments in the francophone, arabophobe, and Luso phone-speaking countries in Africa. However, these findings create a strong starting point for the future expansion of the study.

Our study utilized the Nursing Now Challenge Challengers’ Committee representative in each country to collect details on the profile of each country and not elected/nominated formal leaders in the various governance structures, which may be viewed as a weakness. However, the strength is that we ensured that each representative gained the data on its country from its governance structures and leadership through in-person meetings, phone calls, and verifying those instruments found online. Furthermore, we conducted a Cinahl [34] literature search on each country to enable us to strengthen the data collected. For countries where more clarity was needed, phone calls were made with some of the nursing and midwifery leaders in the relevant countries.

## Supplementary Information

**Additional file 1.**

## Data Availability

'The tools and data set for this study are available upon reasonable request from the corresponding author.
