# Supplementary material for: An overview of nursing and midwifery leadership, governance structures, and instruments in Africa
Source: BMC Nurs. 2023 May 18;22:168. doi: 10.1186/s12912-023-01336-3 (PMC10191683; doi:10.1186/s12912-023-01336-3)
Supplement: Supplementary file 1 — Additional file 1. [file 12912_2023_1336_MOESM1_ESM.docx]

**Questionnaire for Mapping Nursing Leadership and Governance Documents in Africa**

**Section A: Governance Structures**

1. Country ……………………………………….

2. Do you have a department of Nursing at your Ministry of Health? Yes, No

3. Do you have a Chief Nurse or Commissioner of Nursing at your Ministry of Health? Yes, No

4. Does a regulatory council or body for nurses exist in your country? Yes, No

If yes, what's its name? ……………………………………………………………………….

Provide a website link ………………

5. Is there training for your nurses at ( tick all that apply)

1. Certificate level 4
2. Diploma Level 5

Bachelors level 6.

1. Masters level 7
2. Ph.D. level 8

6. Is there a body that oversees the quality of nursing education at various levels and sets exams in your country? yes No

If Yes, what is its name?

Provide web link……………………

7. Do you have a nursing association/federation that all nurses in your country should be part of?

Yes, No

If yes, what is its name?.......................................................................

Is it active in influencing policy development and operationalization? Yes / No /Don’t Know

8. Does your country have a nursing and / OR midwifery labor union? ( A body to advocate for your rights)

If yes, what is its name?......................................................................

9. Is there any mandatory leadership training and education for all nurse leaders in your country?

Do you think it is relevant to have it? Give your reasons

**Details on Various Nursing Policy and leadership Documents**

***Definition of each of the Documents***

**Code of Conduct and Ethics for Nurses*:*** A [code](https://www.collinsdictionary.com/dictionary/english/code) conduct is a set of written rules or norms which explains how people working in a particular profession (nurses) should behave. Many times this is given as a hard copy at the various nurses and midwives councils in Africa.

**Scope of practice:** Highlights the role of each nursing cadre in a particular country. Describes the procedures, actions, and processes that a nurse is permitted to undertake in keeping with the terms of their professional license, education, experience, competency, and laws of the country.

**Schemes of service:** An agreed, defined a guide to the deployment and roles of nurses in various health care organizations.

**Nursing policy:** guides the various sectors of nursing that include service and health care, education, regulation, research, ethical conduct, quality assurance. The nursing policy is a set of overarching principles and goals that advise and guide how nursing care is delivered and accessed. A nursing policy can be established at a national or state level, and then applied in regional areas of specific hospitals and clinical/health services.

**Strategic plan:** A strategic plan guides the implementation of the policy within a given time frame. Strategic planning is the process in which leaders in government, organizations, and professions set out a vision for the future and identify goals and objectives to be achieved. The process also includes establishing the sequence in which those goals should fall so that the vision can be operationalized.

| Nursing Governance Documents | Does this exist?  Yes No | If yes, when was it developed | When was it revised | What is the name of this document | Upload PDF | Is it effectively operational on the ground, if yes how | Is the document available to the public? | Mention and insert a link to where you obtained this document |
| --- | --- | --- | --- | --- | --- | --- | --- | --- |
| Nursing Policy |  |  |  |  |  |  |  |  |
| Nursing Strategic plan |  |  |  |  |  |  |  |  |
| Nursing Schemes of Service |  |  |  |  |  |  |  |  |
| Nursing Scope of Practice |  |  |  |  |  |  |  |  |
| Code of Conduct and Ethics for Nurses |  |  |  |  |  |  |  |  |

10. Which of the above policy documents don’t exist in your country?

Why don’t they exist?

11. What ideas/ strategies do you have to sustainably develop and implement the missing governance documents listed above in your country?

12. In your country, is there a separate profession of midwifery and a separate profession of nursing, or are they the same? Select from the options

- Nursing and Midwifery are covered by one profession
- Nursing and Midwifery are separate professions
- Don’t know

13. In relation to the governance, leadership, and regulation of the nursing profession and the midwifery profession in your country, are the two professions managed, led, and regulated by the same government, official, and governance bodies, or is each profession run separately? Please select :

- Governance, leadership, and regulation of nursing and midwifery is overseen by the same bodies
- The two professions of nursing and midwifery are governed, led, and regulated by different official bodies.
- Don’t know

14. At the front line ( clinical health care in the wards) in the various hospitals , there is no difference in roles for certificate, diploma and bachelors trained nurse. All nurses provide the same care and have the same roles with no differentiation according to level of training.

Yes, No

15. Is there a clear set differentiation in terms of scope of roles for what a certificate , diploma or Bachelors nurse can do during hospital care ?

Yes No
